# Electric-field control of ferromagnetism through oxygen ion gating

**DOI:** 10.1038/s41467-017-02359-6

**Published:** 2017-12-18

**Authors:** Hao-Bo Li, Nianpeng Lu, Qinghua Zhang, Yujia Wang, Deqiang Feng, Tianzhe Chen, Shuzhen Yang, Zheng Duan, Zhuolu Li, Yujun Shi, Weichao Wang, Wei-Hua Wang, Kui Jin, Hui Liu, Jing Ma, Lin Gu, Cewen Nan, Pu Yu

**Affiliations:** 1 State Key Laboratory of Low Dimensional Quantum Physics and Department of Physics, Tsinghua University, 100084 Beijing, China; 2 Institute of Physics, Chinese Academy of Science, 100190 Beijing, China; 3 State Key Lab of New Ceramics and Fine Processing, School of Materials Science and Engineering, Tsinghua University, 100084 Beijing, China; 4 Department of Electronic Science and Engineering, Nankai University, 300071 Tianjin, China; 5 Collaborative Innovation Center of Quantum Matter, Beijing, 100084 China; 6 School of Physical Sciences, University of Chinese Academy of Sciences, 100049 Beijing, China; 7 RIKEN Center for Emergent Matter Science (CEMS), Saitama, 351-0198 Japan

## Abstract

Electric-field-driven oxygen ion evolution in the metal/oxide heterostructures emerges as an effective approach to achieve the electric-field control of ferromagnetism. However, the involved redox reaction of the metal layer typically requires extended operation time and elevated temperature condition, which greatly hinders its practical applications. Here, we achieve reversible sub-millisecond and room-temperature electric-field control of ferromagnetism in the Co layer of a Co/SrCoO_2.5_ system accompanied by bipolar resistance switching. In contrast to the previously reported redox reaction scenario, the oxygen ion evolution occurs only within the SrCoO_2.5_ layer, which serves as an oxygen ion gating layer, leading to modulation of the interfacial oxygen stoichiometry and magnetic state. This work identifies a simple and effective pathway to realize the electric-field control of ferromagnetism at room temperature, and may lead to applications that take advantage of both the resistance switching and magnetoelectric coupling.

## Abstract (web summary)

It has been suggested that the magnetic properties of metal layers using reversible redox reactions could form the basis of memory devices but this requires fast electric control to be practical. Here the authors demonstrate this on sub-millisecond timescales in a metal–oxide heterostructure.

## Introduction

Electric-field control of magnetism, i.e. magnetoelectric coupling, forms one of the key approaches to achieve next generation high-speed and low-power spintronic devices^1–6^. Although the mutual manipulations of both spin and charge degrees of freedom, i.e., electric-field control of magnetization and the magnetic-field control of electric polarization, have been successfully accomplished in a large group of single-phase multiferroic materials^7^, their practical applications are still hindered due to the low transition temperature and weak coupling strength^8,9^. Hence finding alternative pathways to achieve room-temperature magnetoelectric coupling remains an important goal and attracts persistent research efforts in the past decade.

Recently, researchers have demonstrated that oxygen ions in oxide materials can be utilized to effectively manipulate the magnetic properties of ferromagnetic metal layers in metal/oxide heterostructures^10–13^, through the electric-field control of reversible redox reactions within the metal layers. However, to facilitate the oxygen ion migration and necessary redox reactions, extended operating times (several seconds or even minutes) and elevated temperature conditions (~100 °C) are typically required^11–13^. Thus, achieving high-speed performance at room temperature remains one of the main challenges before such simple architectures can be readily adopted in modern semiconductor technologies.

Here, we achieve room temperature, fast (~0.2 ms) electric-field control of the magnetic state in a Co/SrCoO_2.5_ heterostructure, accompanied with non-volatile bipolar resistance switching. The response time (intrinsic speed of the resistance switch) is about four orders of magnitude faster than previous reports in Co/oxide (GdO_*x*_) devices^11–13^, which is attributed to the fact that the oxygen ion evolution is confined within only the SrCoO_2.5_ layer with excellent oxygen ion mobility^14–16^. The electric-field-controlled oxygen evolution leads to oxygen ion accumulation (gating) at the interface, in the same manner as the conventional charge gating device, and as a consequence, the interfacial oxygen contents modulate the magnetic interaction within the Co surface layer and eventually results in the observed magnetoelectric coupling. Our result thus identifies an accessible and efficient strategy to realize high-speed room-temperature magnetoelectric coupling, and furthermore, the hybridization between the magnetoelectric coupling and the accompanying resistance switching effect provides opportunities to design multifunctional iontronic and spintronic devices compatible with modern semiconductor technologies.

## Results

### Bipolar resistance switch across the Co/SrCoO_2.5_ interface

The brownmillerite SrCoO_2.5_ (SCO) was chosen as the oxide layer in the current study, because of its superior oxygen ion mobility through the natural-ordered oxygen vacancy channels^14–16^. High-quality SrCoO_2.5_ thin films were epitaxially grown on conducting 0.5% Nb:doped SrTiO_3_ (001) substrate with the pulsed laser deposition method. The coherent crystalline quality, as well as the atomically flat topography, were evaluated by the X-ray diffraction (XRD) and atomic force microscopy (AFM) measurements (Supplementary Figs. 1 and 2), which were crucial to the subsequent growth of high-quality Co layers.

**Fig. 1:**
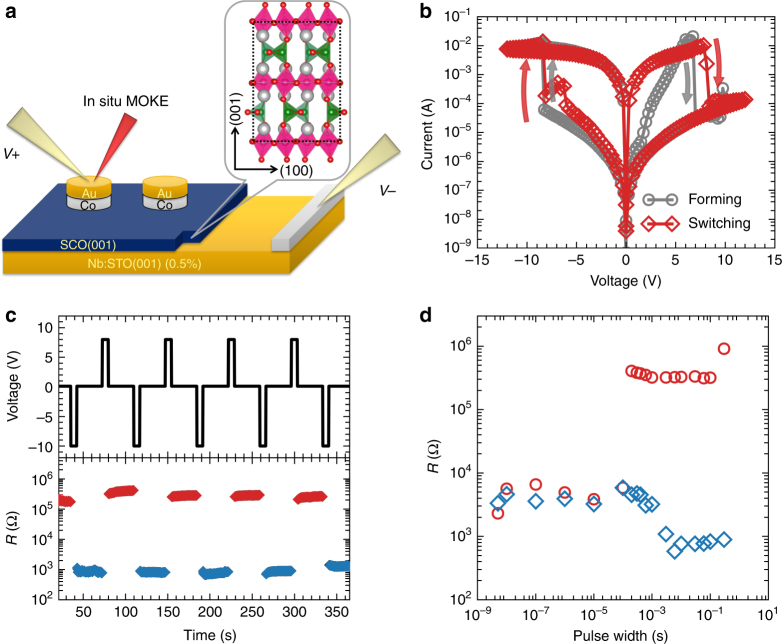
Device configuration and resistance switch in Co/SrCoO_2.5_. **a** Schematic diagram of the device configuration with the Co/SrCoO_2.5_ heterostructure for the in situ resistance switch and MOKE measurements. The electrodes are fabricated into round shape with diameter from 20 to 200 μm. The inset illustrates the crystalline model of SrCoO_2.5_, where the silver and red balls represent the Sr and O atoms, respectively. The Co–O tetrahedral and octahedral ligands are labelled by green and pink polyhedra, respectively. **b** *I*–*V* characteristic curves of the device showing bipolar resistance switch behavior. **c** Memory repetition action based on the bipolar resistance switch. The upper panel shows the pulse sequence, while the lower panel shows the corresponding resistance during the reading. The red and navy blue dots represent the readout of the high-resistance states and low-resistance states, respectively. The reading voltage was fixed at +0.1 V. **d** Resistance switching dynamics with variable pulse durations, where circles and diamonds demonstrate the resistance after positive and negative pulses, respectively

Figure 1a presents a schematic diagram of the device geometry employed in the current study, in which the Co pads were deposited on top of SrCoO_2.5_ using ion-beam-assisted sputtering deposition and a thin layer of Au (or Pt) was fabricated as the capping layer to prevent the Co layer from being oxidized from the surface. During the measurement, the gate voltage was applied between the top Au/Co layer and the bottom conducting Nb:SrTiO_3_ substrate and the SrCoO_2.5_ layers serve as the switching media (i.e., the oxygen ion gate). Figure 1b shows the current–voltage (*I*–*V*) curves of a 6 nm Co device with a characteristic bipolar resistance switch behavior. After the first forming cycle (Supplementary Fig. 3), the resistance of the device can be selectively switched back and forth between the high-resistance state (HRS) and LRS under positive and negative biases, respectively (Fig. 1d), which forms a prototype non-volatile resistive memory (Supplementary Fig. 4). Furthermore, the resistance switch possesses an excellent dynamic response and can be reproducibly and reversibly controlled by the pulsed voltage with duration as short as 0.2 ms (Fig. 1c, d), which can be attributed to the intrinsic oxygen diffusion barrier in SrCoO_2.5_ (see Supplementary Notes 1 and 2).

### Room-temperature electric-field control of ferromagnetism

**Fig. 2:**
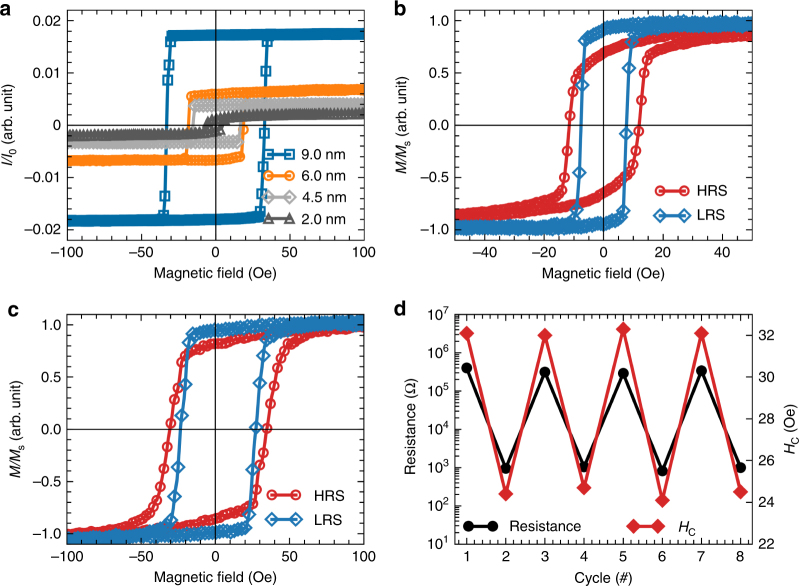
Room-temperature electric-field control of magnetism. **a** Longitudinal Kerr signals of pristine Co/SrCoO_2.5_ devices with different Co thicknesses. **b**, **c** Comparison of the room-temperature magnetic hysteresis loops of Co/SrCoO_2.5_ devices at high-resistance (red) and low-resistance (navy blue) states with the thickness of Co layer at **b** 2.0 nm and **c** 6.0 nm. **d** Reversible modulation of the magnetic coercive field for ~6 nm Co sample with different resistance states. The switching voltages are +8 and −10 V with a duration of 0.6 ms for the HRS and the LRS, respectively. The pads with diameter of ~80 μm were chosen for the studies, and the distance between the tungsten probe and the laser spot was set at ~20 μm

The manipulation between the HRS and the LRS is also accompanied with an intriguing magnetoelectric coupling resulting from the modulation of the magnetic properties of the Co layer. To identify that, we have carried out in situ room-temperature longitudinal magneto-optic Kerr effect (MOKE) measurements during the resistance switch. In the pristine state (Fig. 2a), both the saturated magnetization and the magnetic coercive field are enhanced with the increasing of Co thickness (*t*), which can be attributed to the magnetic relaxation effect of the sputtered polycrystalline Co layer with nanoparticles and clusters^17^. Interestingly, when the device is switched between different resistance states, an accompanying modulation of both the coercive field, as well as the remnant magnetization is observed, in which the high resistant state exhibits larger coercive field and less remnant magnetization as compared with those of the low resistant state (Fig. 2b). To further elaborate the observed magnetoelectric coupling, we define the coercive-field modulation efficiency as ($\eta = \frac{{H_{\rm{C}}^{{\rm{HRS}}} - H_{\rm{C}}^{{\rm{LRS}}}}}{{H_{\rm {C}}^{{\rm {HRS}}}}}$), which reaches about 30% for ~2 nm Co device. With the increase of Co layer thickness to ~6 nm, the efficiency *η* drops quickly to 17% and the modulation of the remnant magnetization is also suppressed (Fig. 2c). When further increasing the thickness of Co layer (~9 nm), the value of *η* decreases to only ~8%, which is hardly detectable with the current measurement approach (see Supplementary Fig. 5). The decay of *η* along with increasing *t* implies that the magnetoelectric coupling may be attributed to modulation at the interface region. Furthermore, the modulation of ferromagnetism is reproducible in the same manner as the resistance switch (Fig. 2d).

### Evidence of the oxygen ion evolution at the interface

**Fig. 3:**
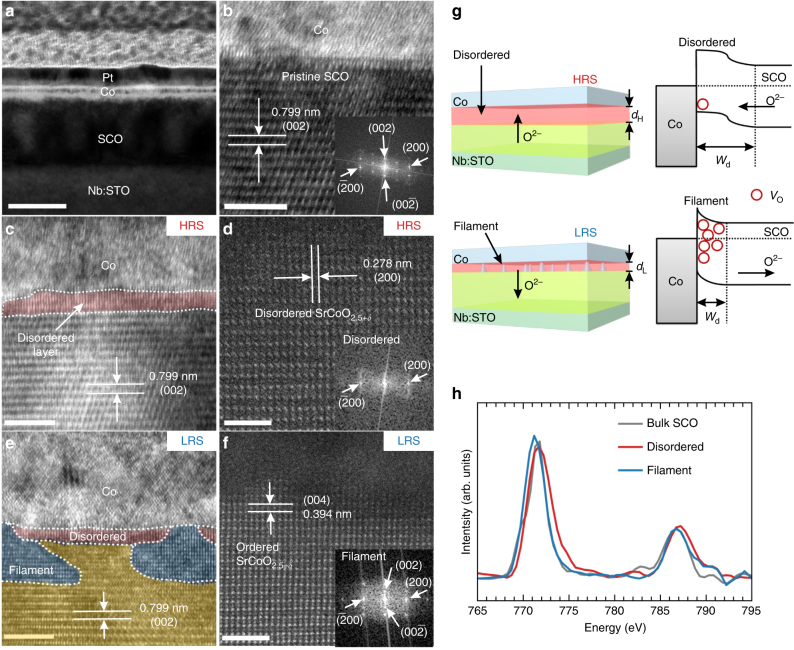
Oxygen ion evolution at Co/SrCoO_2.5_ interfaces. **a** Cross-section low-resolution TEM image of the device structure, in which the scale bar is 50 nm. HRTEM image of Co/SrCoO_2.5_ heterointerface at (**b**) the pristine state and (**c**) the high-resistance state. The disordered SrCoO_2.5+*δ*_ region could be clearly identified as compared with the pristine state, as shown in the magnified image (**d**). **e** HRTEM image of the Co/SrCoO_2.5_ interface in the low-resistance state with the observation of conducting filaments (highlighted by the blue areas). The red and yellow areas denote the remaining disordered and pristine regions, respectively. **f** Detailed crystalline structure at the conducting filament, which are composed of ordered SrCoO_2.5−*δ*_ with pronounced oxygen vacancies (*V*_O_). The insets of (**b**), (**d**), and (**f**) show the diffraction patterns obtained by local FFT. **g** Schematic diagram of the oxygen ion evolution with the corresponding band alignments at the heterointerface. **h** Comparison of Co *L*-edge EELS spectra of the disordered area and the conducting filament at the interface, as well as the bulk region of the SrCoO_2.5_ layer. The *L*_3_ peak values of the filaments, pristine and disordered regions are 771.2, 771.5, and 771.7 eV, respectively. The scale bar indicates 5 nm in (**b**), (**c**) and (**e)** and 2 nm in (**d**) and (**f**)

Knowing that the resistance switch strongly depends on the oxygen evolution at the interface, we deduce that the observed room-temperature magnetoelectric coupling is also closely correlated with the oxygen ion evolution at the heterointerface. To acquire directly the interface structural properties, cross-section transmission electron microscopy (TEM) measurements were performed for samples in pristine state, as well as in the HRS and the LRS. As shown in Fig. 3a, the polycrystalline Co layer forms sharp interface with the SrCoO_2.5_ layer for the pristine samples, and there is no interface oxygen diffusion as confirmed by the fact that the interfacial SrCoO_2.5_ has the same lattice structure as that of the interior layers (Fig. 3b). However, when the device is switched into the HRS under positive bias, the SrCoO_2.5_ layer forms a disordered layer at the interface, in which the featured brownmillerite superstructure stripes disappear (Fig. 3c), as further confirmed by the high-resolution high-angle angular dark-field (HAADF) TEM images and the blurred fast Fourier transform (FFT) pattern (Fig. 3d). On the other hand, when the device is switched to the LRS, the interfacial disordered layer becomes thinner and some regions are changed back to the ordered phase (as shown in Fig. 3e) with the recovery of the diffraction spot in the FFT pattern (Fig. 3f). These features are also clearly verified by extended TEM images of multiple devices as shown in Supplementary Fig. 6. This interesting structural modulation can be understood based on the electric-field-controlled oxygen immigration, as proposed in Fig. 3g. For positive bias voltage, the electric-field drives the negatively charged oxygen ions (O^2−^) towards the Co/SrCoO_2.5_ interface, leading to the formation of the oxygen-rich disordered phase (SrCoO_2.5+*δ*_) with layer thickness of *d*_H_ near the interface. This structural modulation is indeed consistent with the previous in situ XRD studies, in which the (002) superstructure peaks gradually vanish alongside the increase of the oxygen content in SrCoO_2.5_^18^. While for the negative bias voltage, the electric-field drives the oxygen ions away from the interface, leading to the reduced thickness of the disordered oxygen-rich region (*d*_L_ < *d*_H_), as well as the creation of ordered SrCoO_2.5−*δ*_ filaments. These filaments can introduce a multi-filamentary-type resistance switch in the system, which is supported by the area-dependent resistance measurements and capacitance measurements (Supplementary Fig. 7 and Note 3).

Noting that SrCoO_2.5_ is supposed to be an *n*-type semiconductor (*ρ* = 10^1^ – 10^2^ Ω cm at 300 K) with oxygen vacancies as carrier donors^19^, the disordered oxygen-rich region (SrCoO_2.5+*δ*_) can be naturally regarded as a barrier, where the carrier density is suppressed due to the annihilation of vacancies. Thus, the electric-field-driven oxygen migration may result in large variation of the oxygen stoichiometry at the interface, where the modulation of both the depletion region thickness (*W*_d_) of the metal/oxide contact and the corresponding *I*–*V* behavior can be expected^20^. These features are revealed by the comparison of the capacitance–voltage (*C*–*V*) and *I*–*V* measurements on different resistant states (shown in Supplementary Figs. 7b and c). As demonstrated in Fig. 3g, the depletion region in the HRS is expanded compared with that of the LRS, since oxygen vacancies are repelled from the interface, forming an O-rich disordered layer, exhibiting inverse-parallel diode like *I*–*V* behavior (Supplementary Fig. 7c). Furthermore, in the LRS, the oxygen vacancies are accumulated at some regions of the interface with recovered superstructure to form the conducting paths (*n*-doped SrCoO_2.5−*δ*_), consequently leading to the quasi-Ohmic *I*–*V* behavior through the conducting filaments (Supplementary Fig. 7c).

The modulations of oxygen ion content at the interface are also supported by the Co *L*-edge electron energy-loss spectroscopy (EELS) studies at different regions as shown in Fig. 3h, which provide an unambiguous experimental evidence for the Co valence state change^21–23^. To provide a quantitative estimation of the Co valence states, the peak intensity ratios of Co *L*_3_/*L*_2_ are calculated for the filaments, bulk SCO and the disordered region, which are 2.92, 2.75, and 2.61, respectively. By comparing with the reference spectra, the Co valence states in these areas are deduced to be +2.8, +3, and +3.1 correspondingly (Supplementary Fig. 8), which is consistent with the energy shifts of the absorption edges. Thus, these results provide clear evidence for the oxygen migration-induced Co valence state modulation at the interface.

Furthermore, as the resistive switch measurement already provides clear evidence for the formation of non-uniformly distributed filaments, it would be of fundamental importance to extend the magnetoelectric coupling study to different locations within the electrode. The measurements reveal that when changing the distance between the laser spot and the tungsten probe from 20 to 100 μm, the magnetic modulation efficiency drops from ~30% to ~0%, as shown in Supplementary Fig. 9. We note that these interesting results are consistent with the inhomogeneous oxygen concentration at the interface as revealed in Supplementary Fig. 10 and Supplementary Note 4. The results can be understood based on the fact that when the electric field is applied through tungsten probe, the oxygen diffusion starts from the probe contact position and then spreads over the other area of the electrode, as evidenced in the similar system of Co/GdO_*x*_ system^12^.

## Discussion

**Fig. 4:**
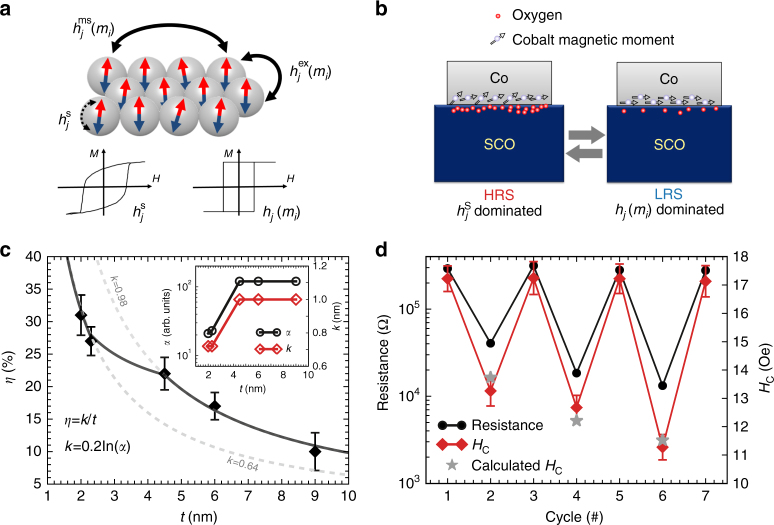
Magnetoelectric coupling via interfacial oxygen ion gating. **a** Schematic diagram of magnetic interactions in granular thin films. The hysteresis loops represent the extreme cases when intrinsic spin flipping $h_j^{\rm s}$ and inter-granular exchanges $h_j\left( {m_i} \right)$ dominate, respectively. **b** Schematic diagram of the magnetoelectric coupling via the segregated oxygen ionic accumulation-induced pinning effect and magnetic anisotropy at the interface, where the intrinsic magnetic flipping field $h_j^{\rm s}$ is enhanced for the high-resistance state (HRS). **c** Co thickness (*t*) dependence of the magnetoelectric coupling for Co/SrCoO_2.5_ heterostructures. *η* is calculated by $(H_{\rm {C}}^{{\rm {HRS}}} - H_{\rm {C}}^{{\rm{LRS}}})/H_{\rm{C}}^{{\rm{HRS}}}$ as mentioned above, while the fitting function is defined as *η* = *k*/*t*. The inset shows the correlation between the coercive field modulation and the ON/OFF ratio of the resistance switch. The error bar is calculated from the standard deviation of results obtained in five different devices. **d** Magnetoelectric coupling with control of oxygen ion gating. The device is set to HRS at the beginning, and then different current limits are set to create the low-resistance state (LRS) with varied resistance values. For each LRS, the coercive field (*H*_C_) was obtained through the measurement of the magnetic hysteresis. The star symbols represent the calculated *H*_C_ values from the ON/OFF ratio during the resistance switch. The error bars indicate the standard deviation from 10 measurements under an identical resistance state

With an understanding of the main mechanism of the resistance switch, we now focus on the observation of the electric-field-controlled oxygen ion accumulation (gating) to explain the origin of the magnetoelectric coupling with two possible mechanisms. We note that for sputtered Co thin films with a granular nature as studied here (shown in Fig. 2a), the magnetic switching field *H*_S_(*j*) can be divided into three interactions: $H_{\rm s}\left( j \right) = h_j^{\rm s} + h_j^{{\rm {ex}}}\left( {m_i} \right) + \,h_j^{{\rm{ms}}}(m_i)$, which are the single grain switching field ($h_j^{\rm s}$), inter-granular interaction ($h_j^{{\rm {ex}}}\left( {m_i} \right)$), and long-range magnetostatic coupling ($h_j^{{\rm{ms}}}(m_i)$), respectively^24^. As illustrated in Fig. 4a, the inter-granular and long-range magnetic couplings lead to a rectangular shape hysteresis loop, while the intrinsic single grain magnetic switching results in a decay of the squareness. Thus, compared with the hysteresis loop in the LRS (Fig. 2b), the decay of squareness in the HRS suggests that the interactions between the granular ($h_j^{{\rm{ex}}}\left( {m_i} \right)$ and $h_j^{{\rm{ms}}}(m_i)$) are reduced, because the oxygen ion adsorption on the Co granular surface suppresses the inter-granular couplings (Fig. 4b)^25^. Meanwhile, the increased magnetic coercive field (i.e. the magnetic flipping barrier) in the HRS can be attributed to the segregated oxygen atoms at the Co interface, which provide an additional pinning effect for the intrinsic magnetic reversal^26^ (Fig. 4b) and subsequently enhance the intrinsic spin flipping barrier. The accumulation of oxygen vacancies at the LRS leads to the enhancement of the inter-granular couplings ($h_j^{{\rm{ex}}}\left( {m_i} \right)$ and $h_j^{{\rm{ms}}}(m_i)$) and suppression of the pinning strength for the intrinsic spin flipping, and consequently the squareness of the hysteresis loop could be restored and the magnetic coercive field is suppressed.

The change of the magnetic anisotropy can also be understood by taking into account the modulation of the interfacial hybridization between O-2*p* and Co-3*d*^27^, in which the tunable interfacial oxygen ions can greatly modify the interfacial Co–O hybridization, and subsequently the magnetic anisotropy of the Co layer^28–30^ (Fig. 4b). For instance, the pristine Au/Co/SrCoO_2.5_ films with ~2 nm Co possess the in-plane surface energy density of about 0.23 erg/cm^2^ (Supplementary Fig. 11), and the surface anisotropy energy density can be changed by about 7% while switching between the HRS and the LRS (Supplementary Note 5). We note that previous calculations show that the switch of Co-3*d* and O-2*p* hybridization can lead up to 0.35 erg/cm^2^ variation for the surface anisotropy^31^, which would naturally explain the clear magnetic modulation through the electric field controlled interface oxygen ion modulation. Furthermore, we notice that a similar electric field-induced magnetic anisotropy change has been achieved experimentally in the MgO/Fe system^32^, which has been attributed to the electric field controlled orbital hybridization between the Fe 3*d* and O 2*p* orbitals^32,33^.

Since the magnetoelectric coupling is strongly correlated with the oxygen stoichiometry within the interface, it would be reasonable to depict the observed decay of efficiency (*η*) along the increase of Co thickness (*t*) as a dimensionless function: $\eta = \frac{k}{t}$, where *k* can be regarded as the nominal effective coupling depth. As summarized in Fig. 4c, the experimental modulation efficiency can be classified into two groups with different nominal effective coupling depths (shown as the dashed lines in Fig. 4c), suggesting different effective coupling strength. Strikingly we have observed that the device resistance ON/OFF current ratio (*α*) (inset of Fig. 4c) follows the same trend as that of the modulation efficiency. Noting that the resistance switch is also correlated with the oxygen ion content at the interface, we can further assess the influence of oxygen ion modulation at the interface by taking *α* into account with a modified function:$\eta = \frac{{k \times \ln \,\alpha }}{t}$. The fitted line according to this improved equation agrees nicely with the experimental results (the solid line of the Fig. 4c). Thus, this correlation between the magnetoelectric coupling and resistance switch provides a pathway to program the magnetoelectric coupling. To demonstrate this, we employed a current limit method^34^ during the resistance switch, which can set the LRS with a tunable resistance value via controlled interfacial oxygen contents (Fig. 4d). As expected, the magnetic coercive field follows the changes of the LRS, and the calculated coercive fields based on parameters obtained from Fig. 4c show excellent agreement with the experimental results.

Finally, we want to remark that the magnetoelectric coupling observed here is conceptually different from previous studies with electric-field-controlled redox reactions within the Co layer^11–13^. Indeed, for the Co/SrCoO_2.5_ system when positive bias voltage is applied for an extended operation time (30–60 s), the oxygen ions would be driven into the Co layer to form CoO_*x*_ (Supplementary Fig. 12), and as a consequence, the ferromagnetic state is changed into superparamagnetic state due to the reduction of the inter-granular coupling with the decrease of magnetic granular volume^25^. However, the formation of CoO_*x*_ results in stronger bonding strength and a higher energy barrier (*E*_a2_) for the oxygen ion diffusion. Indeed, our device is locked into the superparamagnetic state and cannot be recovered to the ferromagnetic state even with the longer operation time (~60 s) and higher voltage (−15 V) employed. On the other hand, the SrCoO_2.5_ layer shows excellent oxygen ion conduction due to its small energy barrier (~0.6 eV along (001) direction)^14^, and as a consequence, the magnetic state can be reversibly and rapidly controlled by the electric-field when the oxygen ion evolution is confined within only the SrCoO_2.5_ layer.

In summary, using the Co/SrCoO_2.5_ heterostructure as a model system, we have demonstrated a strategy to achieve room-temperature magnetoelectric coupling by using oxygen ion gating at the interface. The current study provides a solid foundation to combine the study of magnetoelectric coupling and resistance switch effects together, with potential for creating multi-functional devices compatible with modern semiconductor technologies.

## Methods

### Growth and characterization of the SrCoO_2.5_ thin films

Epitaxial SrCoO_2.5_ thin films with the thickness of 40 nm were grown on conducting Nb:SrTiO_3_ (001) (0.5%) substrate using a home-built pulsed laser deposition system. The growth conditions were optimized at the temperature of 750 °C with the oxygen environment of 100  mTorr. The laser energy (KrF, *λ* = 248 nm) was fixed at 1.2 J/cm^2^ with the repetition rate of 2 Hz. After the growth, the samples were cooled down to room temperature at the cooling rate of 7 °C/min with the growth pressure to avoid the over-oxidation. The crystalline structure and surface morphology of the SrCoO_2.5_ thin films were characterized by XRD (Rigaku, Smartlab) and AFM (Asylum Research), respectively.

### Device fabrication and characterization

The ferromagnetic metal Co (2–10 nm) electrodes with diameters of 20–200 μm were deposited on the patterned SrCoO_2.5_ thin films by ion-beam-assisted sputtering method. A thin Au (or Pt) layer (~3 nm) was deposited in situ on top of Co layers to prevent the oxidation. The transport measurements were performed with the variable-temperature probe station (Advanced Research Systems Inc.) equipped with precision source/measure unit (Agilent B2902A), waveform generator (Agilent 33600A) and LCR meter (Agilent E4980). The electrical contacts were fixed on the Au (or Pt) capping layer with a tungsten microprobe as top electrode (TE) and on the conducting Nb:SrTiO_3_ (0.5%) substrate with the silver paste as bottom electrode (BE).

### Magnetoelectric coupling measurement

In situ longitudinal MOKE measurements were carried out with the NanoMOKE3 system (Quantum Design) using a 532 nm diode laser attenuated to 0.5 mW. The sample was attached to a home-built aluminum holder with external electrodes to link with the same instruments adopted in resistance switch measurements. Then platinum wire was fixed on the BE with adhesive silver paint and the TE was contacted with the same type stiff tungsten microprobe as we used in the probe station to apply the gating voltage and trigger the resistance switch. During these measurements (except the data presented in Supplementary Fig. 10), the distance between the laser spot and the tungsten probe was kept at ~20 μm.

### Transmission electron microscopy

Thin samples for TEM were prepared using focused ion beam (FIB) method and the capping layer is replaced by ~20 nm Pt in order to enhance the compatibility with the Pt protection created in FIB process. They were thinned down to 100 nm thickness at an accelerating voltage of 30 kV with a decreasing current from the maximum 2.5 nA, followed by fine polish at an accelerating voltage of 2 kV with a small current of 40 pA. The high-resolution TEM (HRTEM) images were obtained on a TECNAI F20 TEM (FEI, Eindhoven, Netherlands) operated at an accelerating voltage of 200 kV. The EELS experiments were carried out with a Gatan spectrometer attached to the TEM at the STEM mode. The convergence semi-angle for the electron probe was 13 mrad and the EELS collection semi-angle was 18.3 mrad. Each spectrum was collected with a step of 0.7 nm and energy channel is 0.5 eV/pixel. For HRTEM images, the corresponding experiments were carried out with the transmission electron microscope equipped with double spherical aberration (Cs) correctors (ARM-200CF, JEOL, Tokyo, Japan).

### Data availability

The datasets generated and analyzed here are available from the corresponding author upon reasonable request.

## Electronic supplementary material

Supplementary Information
