# Publisher Correction: Electric-field control of ferromagnetism through oxygen ion gating

**DOI:** 10.1038/s41467-018-02960-3

**Published:** 2018-02-05

**Authors:** Hao-Bo Li, Nianpeng Lu, Qinghua Zhang, Yujia Wang, Deqiang Feng, Tianzhe Chen, Shuzhen Yang, Zheng Duan, Zhuolu Li, Yujun Shi, Weichao Wang, Wei-Hua Wang, Kui Jin, Hui Liu, Jing Ma, Lin Gu, Cewen Nan, Pu Yu

**Affiliations:** 1 State Key Laboratory of Low Dimensional Quantum Physics and Department of Physics, Tsinghua University, 100084 Beijing, China; 2 Institute of Physics, Chinese Academy of Science, 100190 Beijing, China; 3 State Key Lab of New Ceramics and Fine Processing, School of Materials Science and Engineering, Tsinghua University, 100084 Beijing, China; 4 Department of Electronic Science and Engineering, Nankai University, 300071 Tianjin, China; 5 Collaborative Innovation Center of Quantum Matter, 100084 Beijing, China; 6 School of Physical Sciences, University of Chinese Academy of Sciences, 100049 Beijing, China; 7 RIKEN Center for Emergent Matter Science (CEMS), 351-0198 Saitama, Japan

Correction to: *Nature Communications* 10.1038/s41467-017-02359-6; published online 18 December 2017

In the original version of this Article, Figs. 4c and 4d contained incorrectly sized error bars. The correct version is:

**Figure 1:**
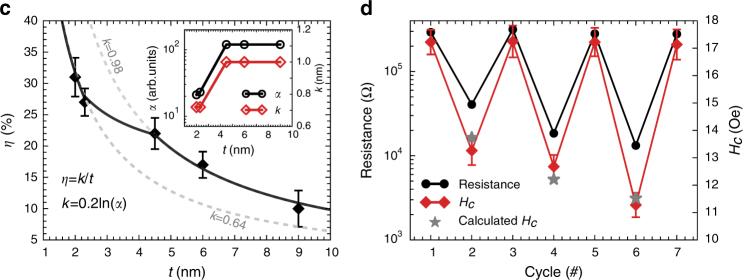

which replaces the previous incorrect version:

**Figure 2:**
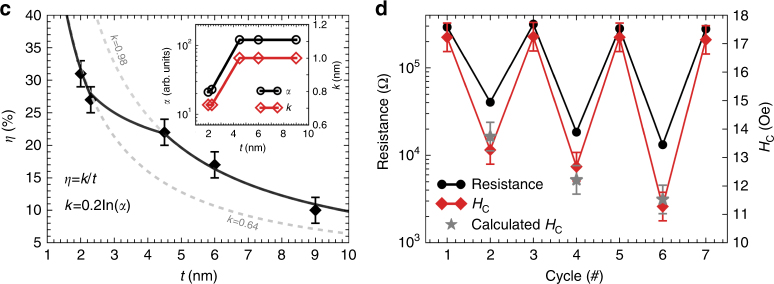

This has now been corrected in both the PDF and HTML versions of the Article.

