# Supplementary material for: Electric-field control of ferromagnetism through oxygen ion gating
Source: Nat Commun. 2017 Dec 18;8:2156. doi: 10.1038/s41467-017-02359-6 (PMC5735161; doi:10.1038/s41467-017-02359-6)
Supplement: Supplementary file 1 — Supplementary Information [file 41467_2017_2359_MOESM1_ESM.pdf]

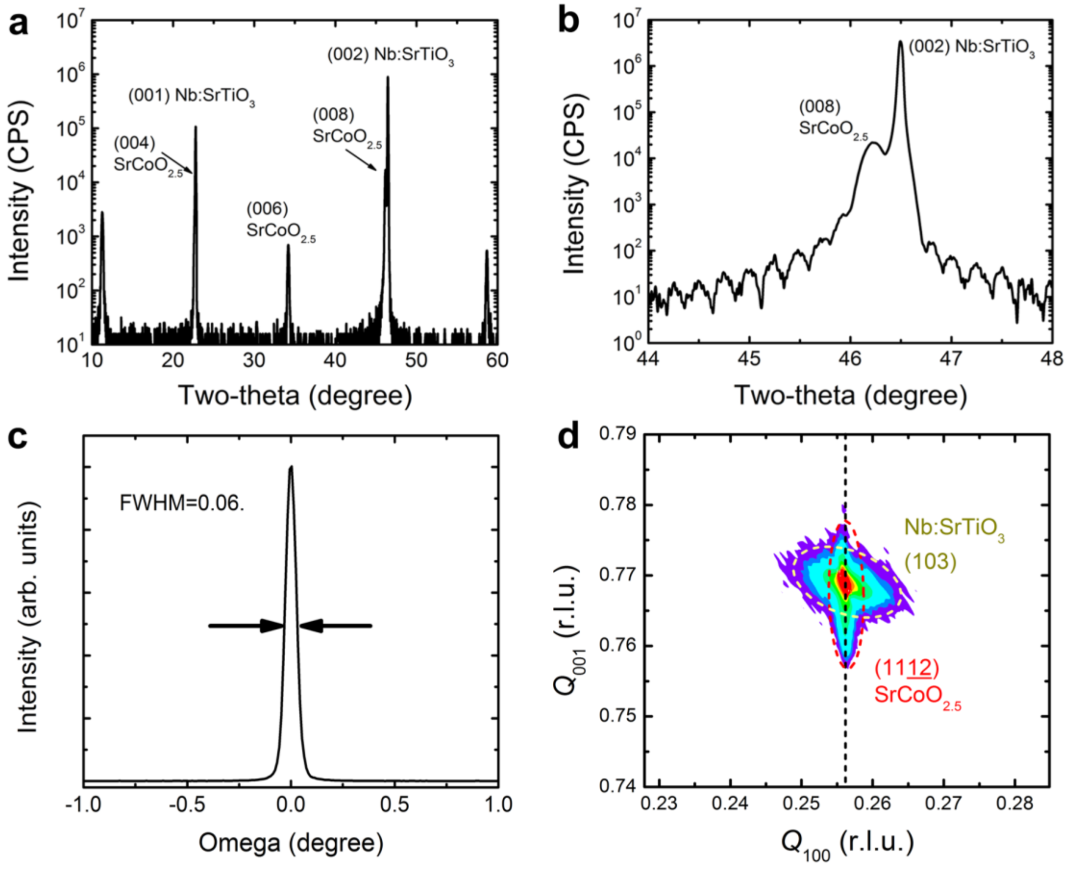

Supplementary Figure 1| Epitaxial growth of high quality $SrCoO_{2.5}$ thin films on 0.5% Nb doped $SrTiO_3$(001) substrate. (**a**) XRD *θ-2θ* scan of $SrCoO_{2.5}$ thin films with thickness of ~40 nm. (**b**) Enlarged image around the (002) peak of the substrate with clear Kiessig fringes around (008) peak of the film, suggesting the high-quality interface and surface structures of the sample. (**c**) Rocking curve of $SrCoO_{2.5}$ (008) peak. The FWHM is ~0.06^{o}, close with that of the substrate, implying a good crystalline quality of the film. (**d**) Reciprocal space mapping around the (103) peak of the substrate, from which it can be concluded that the film is coherently grown on the substrate without in-plane strain relaxation.

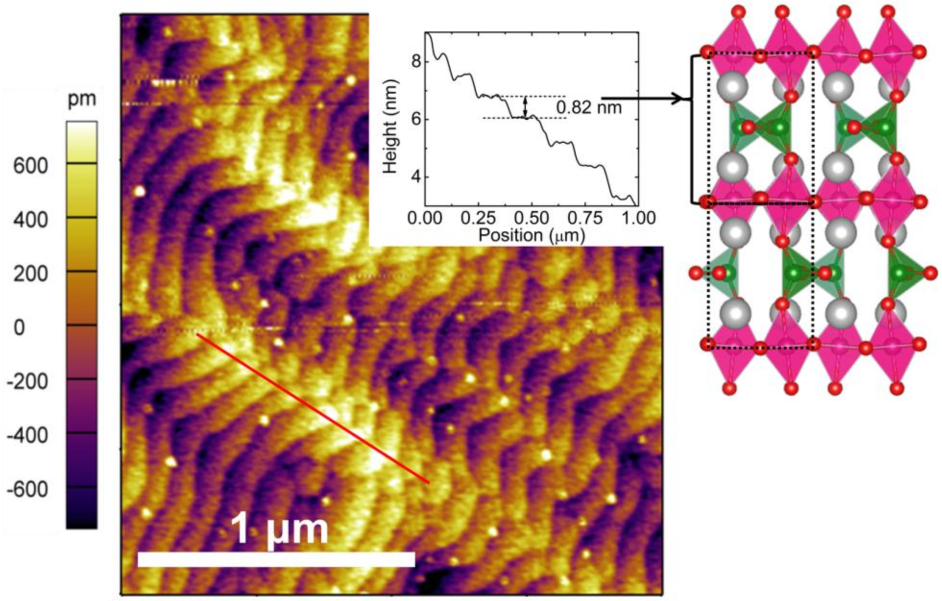

Supplementary Figure 2 | Typical surface morphology of the $SrCoO_{2.5}$ thin film grown on Nb: $SrTiO_3$(001) substrate. The average roughness of the thin film is about 0.33 nm and the atomic terrace can be clearly identified. The inset shows the line scan of atomic terrace as denoted by the red line and the average height of the atomic terrace is about ~0.82 nm, which is about the height of two pseudocubic unit cells of $SrCoO_{2.5}$ with the oxygen tetrahedral and octahedral layers.

Supplementary Note 1. Forming process for the resistance switch.

We note that the forming procedure is relating with the creation of defects in the pristine oxides. A fine scan of the forming process is performed as shown in **Supplementary Figure 3**, where the slope of the Log-Log fitting increases suddenly from 1 to 10 at about 1 V during the forming process, which can be attributed to the creation of substantial defect concentration during forming. Naturally, the defect formation and redistribution would greatly modify the conducting mechanism through the Co and $SrCoO_{2.5}$ interface. We note that the pristine $SrCoO_{2.5}$ possesses an ordered oxygen vacancy channel, which ensures a pronounced oxygen diffusion coefficient. Thus, with the application of electric-field during the forming process, the oxygen ions in bulk would diffuse towards the interfacial layer and lead to the formation of interfacial oxygen-rich disordered regions. Thus, the high resistance state (HRS) is an intrinsically disordered system with large amounts of defects, in which the resistance can be notably smaller than the pristine Co/$SrCoO_{2.5}$ state (**Supplementary Figure 4**). We would like point out that similar changes have been widely discovered in other resistance switching systems as well[1,2]. We further carried out controlled experiments with the voltage scan stopped right before the HRS is fully established (cycle 1 in **Supplementary Figure 3**). The result shows that the formed conducting state is not stable, and recovers back to the insulating pristine state when the voltage is turned off. This result can be understood with the facts that the oxygen-rich disordered region formed during the forming process are still in the transition state at the early stage. Hence once the applied field is gradually removed, the resistance state will be relaxed back to the pristine state. However, if the equilibrium state is established with higher voltage (cycle 2 in **Supplementary Figure 3**), the resistance state would be non-volatile, forming the intriguing property of the resistance switch.

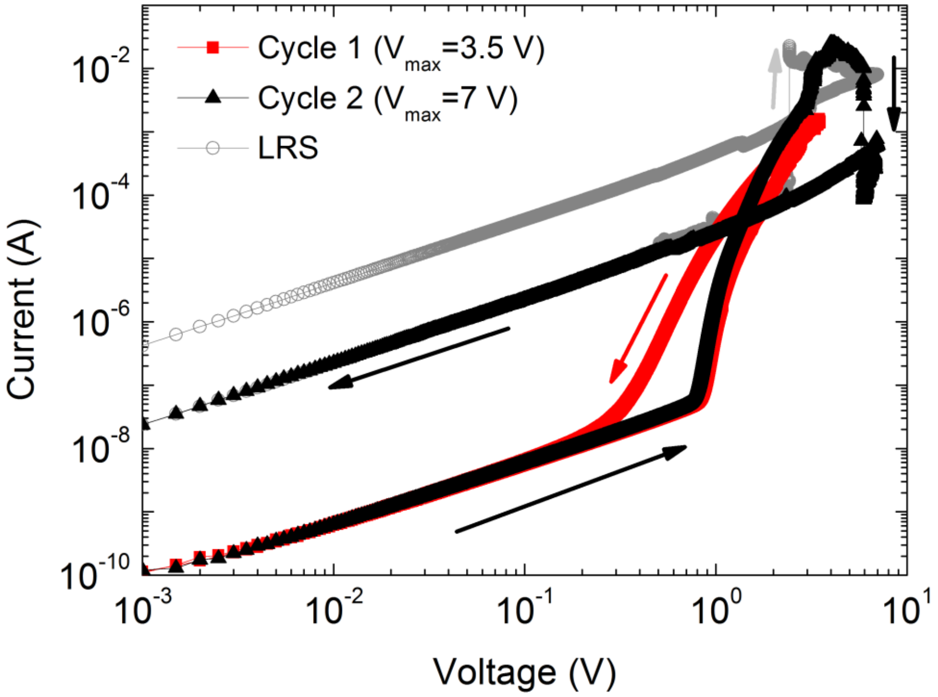

Supplementary Figure 3 | Characteristic I-V curves during forming process with maximum voltage of +3.5 V and +7 V. When positive bias is applied on Co/SrCoO2.5 structures, the disordered layer created during the forming process can be regarded as a self-constructed asymmetric oxide-based RRAM cell. The cycle 1 demonstrates the I-V behavior when the disordered layer is not fully established. The result shows that the formed conducting state is not stable, and recovers back to the insulating pristine state when the voltage is turned off. This phenomenon can be understood with the facts that the oxygen-rich disordered region formed during the forming process are still in the transition state at the early stage. Hence once the applied field is gradually removed, the resistance state will be relaxed back to the pristine state. However, if the equilibrium state is established with higher voltage (cycle 2), the resistance state would be non-volatile, forming the intriguing property of the resistance switch.

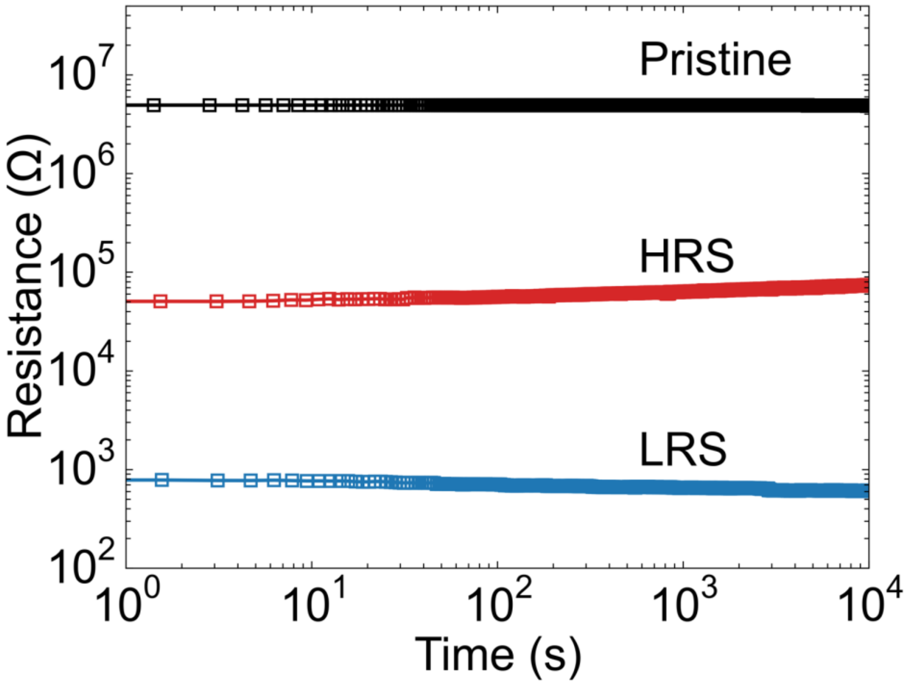

Supplementary Figure 4 | Retention test of the bipolar resistance switch device.

Measurements were performed at room-temperature with the reading voltage of 0.1 V.

Supplementary Note 2. Intrinsic oxygen diffusion barrier determined switching speed

We note that the formation of the disordered layer and the filaments can be related with the oxygen migration, and thus we speculate that the switching speed might be attributed to the intrinsic oxygen ionic conductivity within the $SrCoO_{2.5}$. According to the Fick's law, the diffusion length (L) can be estimated as[3]:

$$L = 2\sqrt{Dt} \quad (1)$$

where D and t are diffusion coefficient and time, respectively. When oxygen ion diffuses along the out-of-plane direction as proposed in the reference work[4] with D=1.2×10^{-12} cm^{-2}s^{-1} at room-temperature[5], it will take about 3×10^{-2} s for oxygen ion to diffuse 4 nm (typical thickness of the disordered layer), which is about two orders of magnitude slower than the experimental results (as shown in **Figure 1d**). However, we note that the diffusion coefficient for during this estimation is the zero-field parameter, which would be further enhanced with the application of electric field. Thus, the experimental resistance switching speed can be increased as well. Nevertheless, this estimation supports the proposal that the current reported switching speed could be limited by the intrinsic ionic mobility of the oxygen in $SrCoO_{2.5}$.

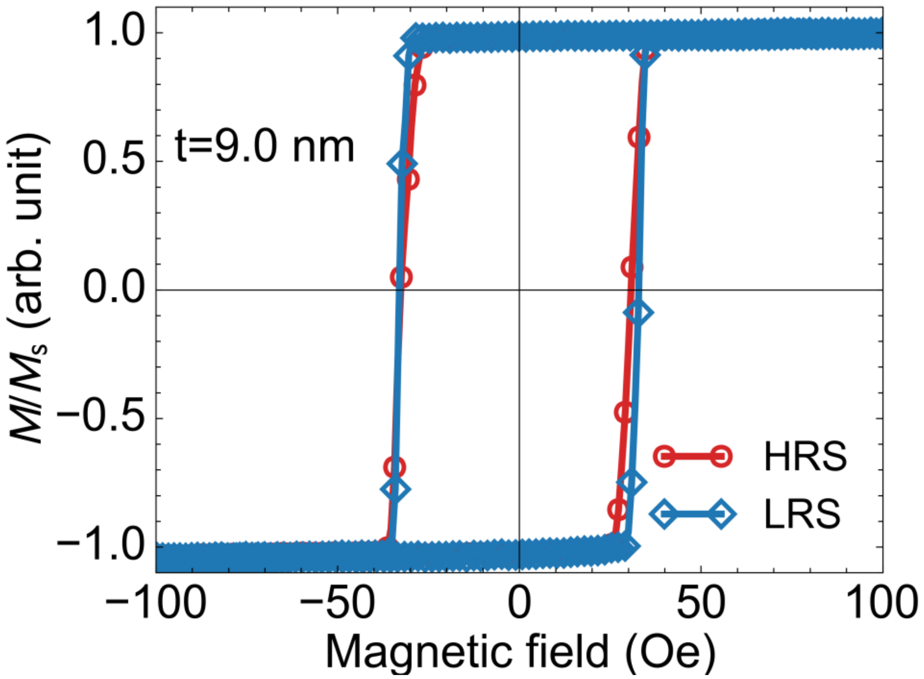

Supplementary Figure 5 | Normalized magnetization curves of the Co /$SrCoO_{2.5}$/Nb:$SrTiO_3$/Ag heterostructure with 9 nm Co layer. The modulation efficiency is dramatically suppressed as the thickness increases.

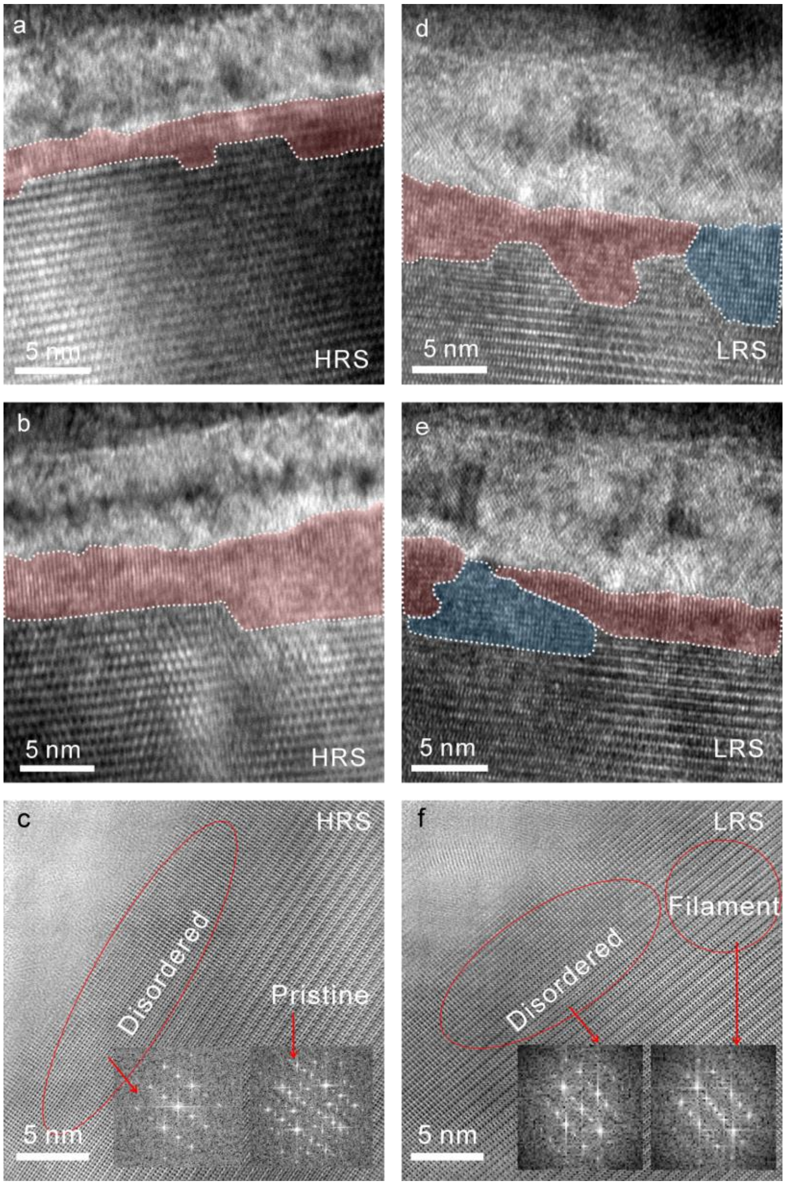

Supplementary Figure 6 | Extended TEM images and high-resolution angular bright-field (ABF) TEM images. TEM images observed at (a, b) HRS and (d, e) LRS with disordered layer and filaments labelled by red and blue areas. ABF images of (c) HRS and (f) LRS, where the superstructure is vanished in HRS as compared with LRS. The structural periodicity is determined with local FFT analysis as shown in the inset of (**c**) and (**f**).

Supplementary Note 3. Inhomogeneous distribution of conducting filaments.

Conventionally, the filamentary and homogeneous resistance switch mechanisms can be distinguished by the area dependence of the resistance values in high resistance and low resistance states[6]. The extreme cases are single filamentary type and homogeneous interfacial type, where the resistance of the former one is independent of the contact area, while the resistance is in linear relationship with the contact area for the latter one[6]. However, current studies demonstrate that many resistance switch systems are dominated by the combination of these two cases[7]. Furthermore, recent numerical calculations and experiment researches demonstrate that the area dependency of the resistance states could also exist in multi-filament resistance switch systems, similar as the behavior of homogenous interfacial type switching[8], in which the slope of the resistance vs. area curves in the Log-Log plot is close to -1. In our experiment, both high resistance and low resistance states display the same linear relationship between the resistance values and the contact areas on Log-Log plots (**Supplementary Figure 7a**), with the slope of about -0.7. This small value of the slope suggests an inhomogeneous distribution of the conduction paths as well as the oxygen-rich disordered region[8]. Indeed, the existences of multiple conduction filaments and inhomogeneous oxygen-rich disordered regions have been confirmed with the HRTEM results on for the LRS (**Figure 3e)** and HRS samples (**Supplementary Figure 10**). Besides, due to the high oxygen ionic mobility within the $SrCoO_{2.5}$ layer, the O^{2-} (V_O) migration leads to not only the formation of the conducting filaments, but also the accompanied dramatic modulation of the Schottky barrier thickness (depletion region), similar as observed in other complex oxide based metal/oxide resistance switch devices[9,10]. The C-V measurements are performed to get further insight of the change of the depletion region during the resistance switch (shown

in **Supplementary Figure 7b**). The C-V curve of the LRS exhibits a high capacitance state (HCS), which corresponds to a narrow depletion region, while the HRS corresponds to a low capacitance state (LCS), suggesting widened depletion region. Furthermore, in high capacitance state, the capacitance drops dramatically to the low capacitance state at about +6 V, consistent with the switching voltage in I-V measurements (see **Figure 1b** in the main text**)**. Furthermore, the I-V curve of the HRS state displays the inverse-parallel diode characteristic, while the LRS shows the quasi-Ohmic behavior, as plotted in **Supplementary Figure 7c,** consistent nicely with the modulation of the depletion region. These results suggest that the depletion region at the interface is significantly modulated by the electric-field induced oxygen ion accumulation (gating) at the interface. We note that the current system can be taken as the analogous to the asymmetric RRAM model system of Pt/Ta_2O_{5-x}/TaO_x/Pt, in which a few nanometer thick Ta_2O_{5-x} layer acts as the function layer with the responsibility to host the conducting filaments under external bias[11]. Accordingly, in the Co/$SrCoO_{2.5}$ structure, the disordered layer created during the forming process can also be regarded as a self-constructed function layer. Such behavior could be attributed to the natural ordered oxygen channels in $SrCoO_{2.5}$, as well as the versatile crystalline structure of brownmillerite $SrCoO_{2.5}$.

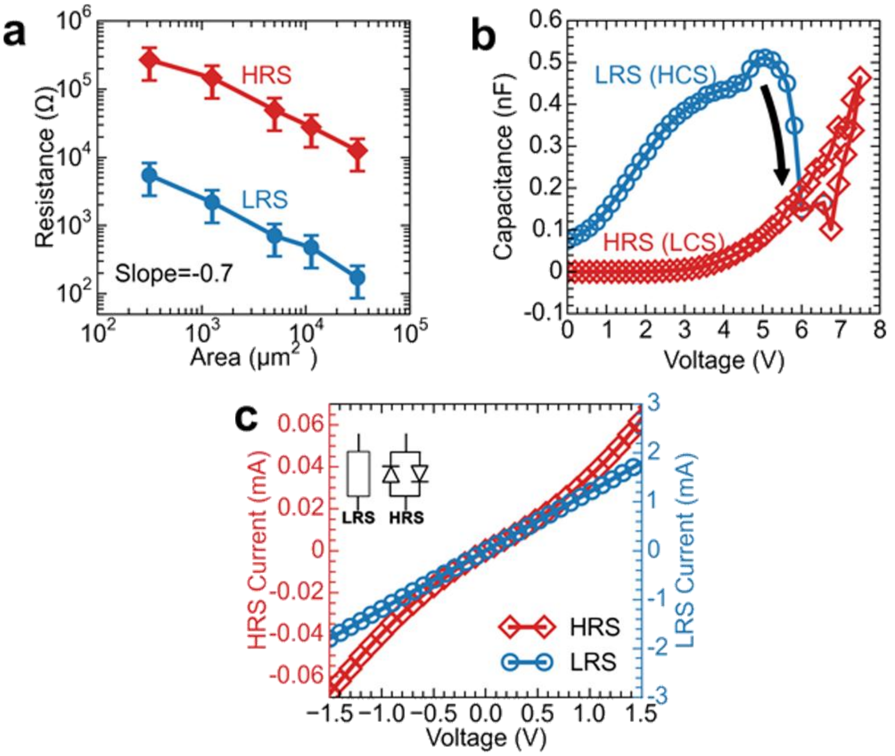

Supplementary Figure 7 | Resistance switch with the combined oxygen evolution controlled multi-filament and homogeneous interface mechanisms. (a) Area dependent study of the resistance values at high and low resistance states. **(b)** Comparison of the C-V measurements for the HRS and LRS at the Co/$SrCoO_{2.5}$ device. AC signal (100 kHz) with the amplitude of 50 mV was employed for the measurement. **(c)** Typical I-V curves for the HRS and LRS. The linear and exponential fittings are corresponding to the quasi-Ohmic and inverse-parallel diodes behaviors at the low resistance and high resistance states respectively.

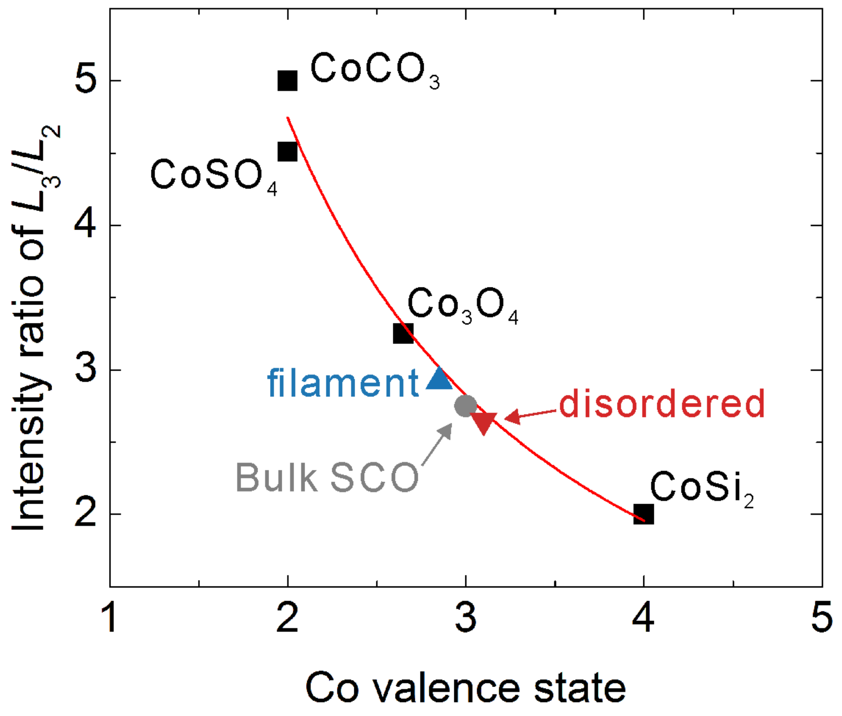

Supplementary Figure 8 | Co valence state assignments by calculating the intensity ratio of L_3/L_2 in EELS data. The data points represented by black squares are extracted from the reference[12]. To quantify the Co valence state at different regions, the ratio between L_3 and L_2 peaks are calculated after the background subtraction, which was proved previously to be a good method to determine the Co valence state[12, 13]. The measured peak ratios of L_3/L_2 from our samples were plotted together with the data at reference samples with Co^{2+}, Co^{3+} and Co^{4+}, where the ratio decreases with the increment of Co valence state. Following this guideline, the Co valence states are estimated as +3.1, +3, and +2.8 for the disordered, bulk and the filament regions respectively.

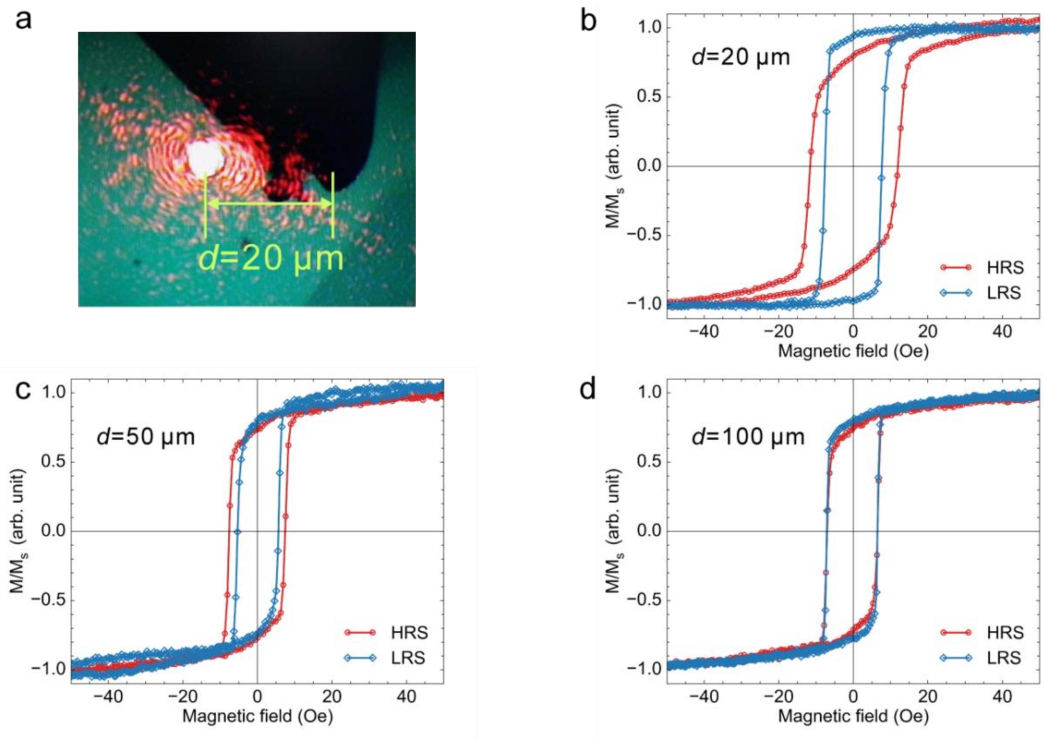

Supplementary Figure 9 | Probe-laser distance dependence for magnetic modulations. (**a**) Image taken during the in-situ MOKE studies, where the red area is the laser spot on the sample and the black object is the tungsten tip. Experimental results obtained at sites (**b**) 20, (**c**) 50 and (**d**) 100 μm away from the probe, respectively. When the distance between the laser spot and the probe is increased from 20 μm to 100 μm, the magnetic modulation efficiency drops from ~30% to 0%. When the measurements performed at different sites with the same distance, we obtained similar magnetic modulation results, indicating that the distance is the key parameter to influence the magnetoelectric coupling.

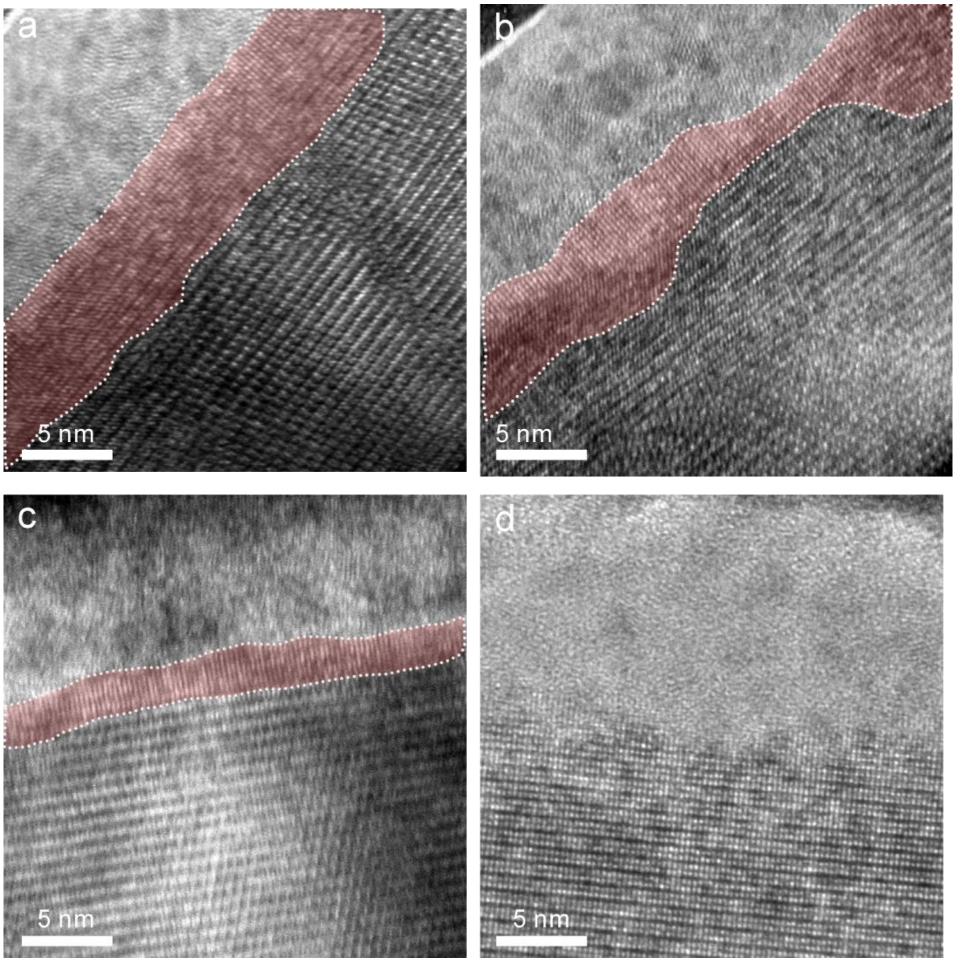

Supplementary Figure 10 | TEM images on samples fabricated from different sites of the same electrode (with the diameter of 200 µm) at HRS. The distances between these sites to the position of the probe are (a) *d*=10 µm, (b) *d*=20 µm, (c) *d*=50 µm and (d) *d*=100 µm, respectively. Red regions outline the disordered structures at HRS.

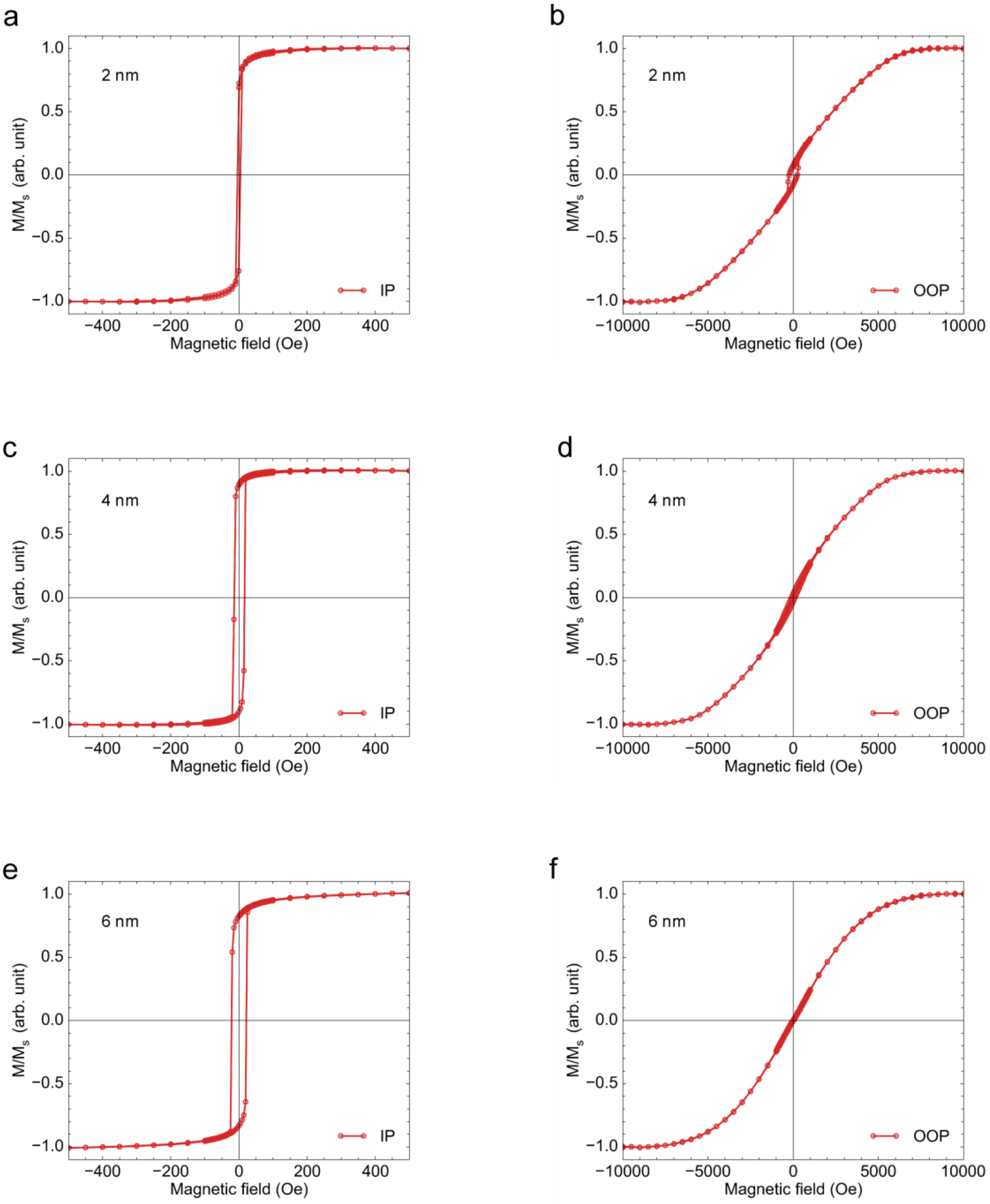

Supplementary Figure 11 | In-plane and out-of-plane M-H curves of as-grown Au/Co/$SrCoO_{2.5}$ thin film with Co thickness of (a, b) 2 nm, (c, d), 4 nm (e, f) and 6 nm, respectively.

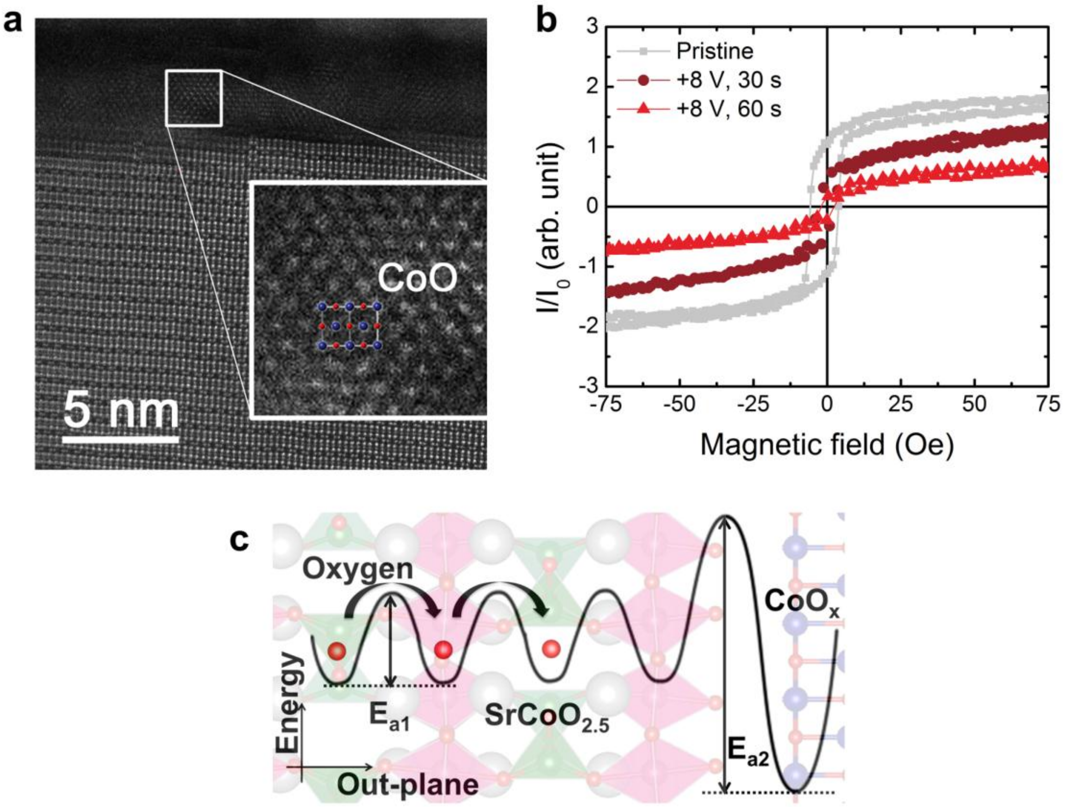

Supplementary Figure 12 | Electric-field induced oxidation of the Co layer. (**a**) TEM measurements when device is biased with positive external voltage for extended operation time. The formation of CoO layers at the interface suggests that the oxygen ions can be driven into the Co layer to form CoO with the application of electric field. (**b**) Evolution of magnetization with the oxidation of the Co layer. The superparamagnetic behavior of the granular system appears as the Co layer is oxidized, which can be attributed to the lowered flipping energy barrier and suppressed inter-granular coupling through oxidation. **(c)** Schematic diagram of energy potential landscape for the oxygen ion migration across the interface. Due to the presence of the naturally ordered oxygen vacancy channels in the $SrCoO_{2.5}$ layer, the potential barrier for oxygen ion diffusion is

relatively smaller, which is in favor of the electric-field controlled oxygen ion migration within the $SrCoO_{2.5}$ layer. However, the large energy barrier between the $SrCoO_{2.5}$ and CoO layers hinders the oxygen ion evolution, and thus to facilitate the diffusion across the interface, elevated temperature condition or extended operation time are desired. In another word, the large difference between the energy barriers within $SrCoO_{2.5}$ bulk and across the interface can confine the oxygen ion evolution only within the $SrCoO_{2.5}$ layer, as revealed by the current study.

Supplementary Note 4. Inhomogeneous oxygen-ion distribution at the interface.

To obtain the direct information at the interface, we have carried out extended TEM studies with different distances from the probe within the same electrode (diameter of 200 μm), as shown in **Supplementary Figure 10.** The disordered layer is defined as the region where superlattice diffraction patterns are destroyed as evaluated by the local FFT analysis. Accordingly, the average thicknesses of the disordered region for 10 μm, 20 μm and 50 μm are 6.1±1.0 nm, 4.8±1.8 nm and 3.2±0.7 nm, respectively, which presents a clear variation of the non-uniform oxygen distribution across the device. More importantly, when the distance is increased to ~100 μm, the disordered region could hardly be resolved at the interface, indicating that the interface remains close to the pristine state despite of the application of the electric field. These results are consistent with the previous studies in Co/GdOx system, where the oxygen ion modulation is pronounced at region close to the probe contact[14]. Finally, it is important to note that, since the pristine state possesses higher resistance than both HRS and LRS, the remained pristine area will not influence the I-V modulation observed in the study.

Supplementary Note 5. Electric field control of the magnetic anisotropy.

To further demonstrate the magnetic modulation during the resistance switch, the Co/SrCoO_x heterostructures were measured with the superconducting quantum interference device (SQUID, Quantum design), from which the typical thickness dependent magnetic anisotropy of polycrystalline Co thin films was deduced. When the thickness is decreased to ~2 nm, the magnetic anisotropy is suppressed with the out-of-plane magnetization enhanced, which is consistent with previous reports on the sputtered polycrystalline Co thin films[15,16]. With increase of Co thickness, the in-plane surface energy densities are estimated as 0.23, 0.80 and 1.51 erg/cm^2 for 2, 4 and 6 nm Co thin films, which agrees nicely with previous reports on Au/Co systems, in which the in-plane magnetic anisotropy is enhanced when Co thickness is increased.

With the knowledge about the magnetic anisotropy in the as-grown Co/SrCo$O_{2.5}$ heterostructure, we further investigated the modulation of the magnetic anisotropy during the resistance switch. When the device was switched from LRS to HRS with the application of the electric field, the in-plane surface anisotropy energy (E_{IP}) can be calculated from MOKE results by assuming a linear relationship between the Kerr ellipticity η_k and the magnetization M[17]:

$$E_{\mathrm{IP}} = -\mu_0 \frac{M_s}{\eta_s} \int_0^{\eta_s} H \, d\eta_k \qquad (2)$$

where μ_0, M_S, H, η_k and η_s are permeability of free space, saturated magnetization, external magnetic field, Kerr ellipticity and saturated Kerr ellipticity, respectively. When the device is switched from HRS to LRS, we define the ratio of the magnetic anisotropy modulation in a similar manner as the H_{C} modulation efficiency η used in the main text,

i.e. $\Delta E_{IP}/(E_{IP,\,HRS})=(E_{IP,HRS}-E_{IP,LRS})/(E_{IP,HRS})$, which is estimated as 7%, i.e. ~0.02 erg/cm^2 for the ~2 nm Co device.

1. Lee, S. B. *et al.* Forming mechanism of the bipolar resistance switching in double-layer memristive nanodevices. *Nanotechnology* **23,** 315202 (2012).
2. Liu, C. Y. Memory effect of sol–gel derived V-doped $SrZrO_3$ thin films. *Thin Solid Films* **494,** 287-290 (2006).
3. Bird, R. B., Stewart, W. E. & Lightfoot, E. N. Transport Phenomena. *John Wiley & Sons.* (1976).
4. Mitra, C., Meyer, T., Lee, H. N., & Reboredo, F. A. Oxygen diffusion pathways in brownmillerite SrCoO2.5: Influence of structure and chemical potential. *J. Chem. Phys.* **141,** 084710 (2014).
5. Mefford, J. T. *et al.* Water electrolysis on $La_{1-x}Sr_xCoO_{3-\delta}$ perovskite electrocatalysts. *Nat. Comm.* **7,** 11053 (2016)
6. Sawa, A. Resistive switching in transition metal oxides. *Mater. Today* **11,** 28-36 (2008).
7. Waser, R., Dittmann, R., Staikov, G. & Szot, K. Redox-based resistive switching memories - nanoionic mechanisms, prospects, and challenges. *Adv. Mater.* **21,** 2632-2663 (2009).
8. Tanaka, H., Kinoshita, K., Yoshihara, M. & Kishida, S. Correlation between filament distribution and resistive switching properties in resistive random access memory consisting of binary transition-metal oxides. *AIP Adv.* **2,** 022141 (2012).
9. Szot, K., Speier, W., Bihlmayer, G. & Waser, R. Switching the electrical resistance of individual dislocations in single-crystalline SrTiO3. *Nat. Mater.* **5,** 312-320 (2006).
10. Sawa, A., Fujii, T., Kawasaki, M. & Tokura, Y. Interface transport properties and resistance switching in perovskite-oxide heterojunctions. *Proc. SPIE* **5932,** 59322C (2005).
11. Lee, M. J. *et al.* A fast, high-endurance and scalable non-volatile memory device made from asymmetric Ta_2O_{5-x}/TaO_{2-x} bilayer structures. *Nat. Mater.* **10,** 625-630 (2011).
12. Wang, Z. L., Yin, J. S., & Jiang, Y. D. EELS analysis of cation valence states and oxygen vacancies in magnetic oxides. *Micron* **31,** 571-580 (2000).
13. Zhao, Y., Feltes, T. E., Regalbuto, J. R., Meyer, R. J. & Klie, R. F. In situ electron energy loss spectroscopy study of metallic Co and Co oxides. *J. Appl. Phys.* **108,** 063704 (2010).
14. Bauer, U. *et al.* Magneto-ionic control of interfacial magnetism. *Nat. Mater.* **14,** 174-181 (2015).
15. Manchon, A. *et al,* Analysis of oxygen induced anisotropy crossover in Pt/Co/MOx trilayers. *J. Appl. Phys.* **104,** 043914 (2008).
16. Rodmacq, B., Manchon, A., Ducruet, C., Auffret, S. & Dieny, B. Influence of thermal annealing on the perpendicular magnetic anisotropy of $Pt/Co/AlO_x$ trilayers. *Phys. Rev. B.***79,** 024423 (2009).
17. Maruyama, T. *et al.* Large voltage-induced magnetic anisotropy change in a few atomic layers of iron, *Nat. Nanotech.* **4,** 158-161 (2009).
